# Testosterone Increases the Emission of Ultrasonic Vocalizations With Different Acoustic Characteristics in Mice

**DOI:** 10.3389/fpsyg.2021.680176

**Published:** 2021-06-25

**Authors:** Takefumi Kikusui, Miku Sonobe, Yuuki Yoshida, Miho Nagasawa, Elodie Ey, Fabrice de Chaumont, Thomas Bourgeron, Kensaku Nomoto, Kazutaka Mogi

**Affiliations:** ^1^Department of Animal Science and Biotechnology, School of Veterinary Medicine, Azabu University, Sagamihara, Japan; ^2^Human Genetics and Cognitive Functions, Institut Pasteur, UMR 3571 CNRS, Université de Paris, Paris, France

**Keywords:** ultrasonic vocalization, mice, masculine behavior, testosterone, syllable complexity

## Abstract

Testosterone masculinizes male sexual behavior through an organizational and activational effects. We previously reported that the emission of ultrasonic vocalizations (USVs) in male mice was dependent on the organizational effects of testosterone; females treated with testosterone in the perinatal and peripubertal periods, but not in adults, had increased USV emissions compared to males. Recently, it was revealed that male USVs have various acoustic characteristics and these variations were related to behavioral interactions with other mice. In this regard, the detailed acoustic characteristic changes induced by testosterone have not been fully elucidated. Here, we revealed that testosterone administered to female and male mice modulated the acoustic characteristics of USVs. There was no clear difference in acoustic characteristics between males and females. Call frequencies were higher in testosterone propionate (TP)-treated males and females compared to control males and females. When the calls were classified into nine types, there was also no distinctive difference between males and females, but TP increased the number of calls with a high frequency, and decreased the number of calls with a low frequency and short duration. The transition analysis by call type revealed that even though there was no statistically significant difference, TP-treated males and females had a similar pattern of transition to control males and females, respectively. Collectively, these results suggest that testosterone treatment can enhance the emission of USVs both in male and female, but the acoustic characteristics of TP-treated females were not the same as those of intact males.

## Introduction

Ultrasonic vocalizations are interesting behavioral phenotypes in rodents. Upon encountering females or female urinary pheromones, male mice emit ultrasonic vocalizations (USVs) (Nyby et al., 1977b). Adult males emit more USVs than adult females (Nyby et al., 1977a), and male USVs also stimulate female sexual function (Asaba et al., 2017; Nomoto et al., 2018). In addition, characteristics of USVs differ among strains; for example, C57/BL6 (B6) males show a higher peak frequency of calls, shorter intervals between calls, and more “jump” calls, whereas BALB/cA males present more harmonic calls (Kikusui et al., 2011). These differences in song characteristics act as social cues that determine the preferences of female mice. In particular, female mice prefer the USVs from different strains of male mice, which implies that USVs are used to avoid inbreeding (Asaba et al., 2014).

Recent studies have revealed that various types of calls are observed in a social context-dependent manner. Sangiamo et al. examined USV emissions of four mice when they were put into a group. They analyzed mice social behavior together with USVs emissions in a spatiotemporal monitoring system and used deep learning methods to classify the calls into 22 types. Calls with lower frequency occurred during aggressive behavior, while calls with higher frequency occurred during escaping behavior (Sangiamo et al., 2020). Ey et al. revealed that mice used call types in a context-specific manner in same-sex pairs. Interestingly, female pairs emitted more USVs than males when encountering the female partner (Ey et al., 2020). Collectively, female mice have the potential to emit USVs, and the emission of USVs also reflects a high level of arousal in social interactions across different contexts (Neunuebel et al., 2015; Ey et al., 2020). In the context of male and female sexual interactions in standard experimental settings, male mice are more aroused and emit more USVs than females (Whitney et al., 1973; Warburton et al., 1989).

Sex differences in USVs emission during sexual interaction depend on the sexual differences in arousal and motivation elicited by sexual cues (Schober and Pfaff, 2007). In this regard, female sexual cues stimulate male sexual arousal and motivation, and the neural circuits regulating USV emissions by female cues have also been described. In females, pheromonal information inhibits USV emission, and functional neuronal circuits for USV emission exist in the female brain (Kimchi et al., 2007). We also reported that while male sexual behavior, such as mounting, is mediated via the vomeronasal neural circuits, USVs emissions induced by chemosignals are mediated predominantly by the main olfactory system (Matsuo et al., 2015), suggesting that these male sexual behaviors are regulated by distinct neural circuits. Recently, we reported that perinatal testosterone propionate (TP) treatment in female mice was necessary to induce comparable numbers of USVs to males, and adult TP treatment was not enough for USVs emission (Kikusui et al., 2020). Therefore, the neural circuits that regulate male mounting behavior and USVs are both masculinized by perinatal testosterone.

In a previous study, the organizational effect of testosterone was assessed by the number of emissions, and call type analysis was not performed. As mentioned above, male mice emitted various types of calls depending on the social context (Ey et al., 2020; Sangiamo et al., 2020). Female and male mice are capable of producing the same acoustic characteristics, instead they vary the acoustic features of USVs based on context; female USVs produced during male/female interactions have different acoustic characteristics to the male USVs (Warren et al., 2018, 2020). A question arises as to whether treatment with testosterone can also masculinize call types induced by encounters with females. Given the previous findings, the neonatal testosterone masculinizes the sexual motivation in female mice, it was proposed that testosterone treated females showed equivalent acoustic characteristics and call types to male mouse in a standard experimental setting. A question has also arisen whether the additive effects of TP induce more masculinized males. In this study, we aimed to address this issue by examining the acoustic characteristics of USVs induced by the treatment of testosterone both in female and male which were treated with TP in perinatal and juvenile periods.

## Materials and Methods

All the vocalization data were acquired in our previous study (Kikusui et al., 2020), and we analyzed the acoustic characteristics of the calls using a state-of-the-art machine learning method (Ey et al., 2020) which was not available at the time of the initial publication. We have described the methods briefly below.

### Animals

C57BL/6J male and female mice were originally obtained from CLEA Japan, Inc. (Shizuoka, Japan). All experimental procedures were approved by the Ethics Committee of Azabu University (# 160303–6). Subject mice were kept in groups of three with individuals of the same sex after weaning.

### Measurement of USVs

The procedures for USV measurement were in line with our previous studies (Asaba et al., 2014; Kikusui et al., 2020). In brief, sexually naive males and females at the age of 8 weeks were singly housed (172 × 240 × 129 mm) 1 week before the USV recording. The cage that contained the subject mouse was placed in a soundproof chamber and an unfamiliar virgin female (8–12 weeks old) was introduced. To control the estrus cycle, the female mice were ovariectomized under isoflurane anesthesia (5%). After ~2 weeks of recovery, ovariectomized female mice were primed with 17β-estradiol and progesterone at concentrations of 20 μg per 0.05 ml and 300 μg per 0.03 ml in corn oil, respectively, at 24 and 48 h before testing (Asaba et al., 2014). USVs were recorded for 5 min using a microphone (CM16/CMPA, Avisoft Bioacoustics, Brandenburg, Germany. Frequency range: 2–200 kHz) and an A/D converter (Avisoft-UltraSoundGate116H, Avisoft Bioacoustics). Although the number of calls from the presenting female mice was limited due to the fact that they were tested in the small cages reported previously (Whitney et al., 1973; Warburton et al., 1989), the calls recorded and analyzed were those emitted by the pair of mice. The recorded calls were as follows: control females (*n* = 7, median 332 calls), control males (*n* = 15, median 522 calls), TP-treated females (*n* = 14, median 614 calls), TP-treated males (*n* = 7, median 1,249 calls); therefore, there were in total 43 mice and 38,957 calls.

### Testosterone Treatments

Testosterone treatments were performed as reported in our previous study (Kikusui et al., 2013, 2020). In brief, female and male mice were treated with TP (Wako Pure Chemicals, Osaka, Japan) during the neonatal and peripubertal periods. For perinatal treatment, pregnant dams were injected with TP on embryonic days (EDs) 15, 16, and 19. Two injections were administered to pups on post-natal (PD) 0 and 2. Control injections were performed with corn oil, and the TP was also dissolved in corn oil. Administration was performed as follows: TP injection in pups, 1.25 μg/0.02 mL, s.c; TP injection in dams, 1.25 μg/0.02 mL, s.c.

On PD21, steroid capsules were surgically implanted, which comprised cholesterol powder (25%, Wako Pure Chemicals, Osaka, Japan) with TP (75%) packed in a 7-mm silicone tube (external diameter 2 mm; internal diameter 1 mm), with silicone at both ends. Four groups were used in the following analysis: control females (*n* = 7), control males (*n* = 15), TP-treated females (*n* = 14), and TP-treated males that received excessive testosterone as compared to control males (*n* = 7).

### Acoustic Characteristics Analysis

The calls were automatically detected using online software developed by Ey et al. (USV detector: https://usv.pasteur.cloud/) (Ey et al., 2020). In brief, background noise was removed by filtering the spectrum data, and then signals were extracted using machine learning. In order to reveal the phonetic properties of calls, the following acoustic characteristics were computed for each USV (Supplementary Table 1): duration (ms), frequency dynamic (Hz), mean frequency (Hz), frequency TV (Hz), delta frequency (Hz; difference between end frequency and start frequency), mean frequency TV (Hz), linearity index, nb of modulations, nb of jumps, minimal frequency (Hz), maximal frequency (Hz), and peak frequency (Hz). These parameters and the number of calls (Voc number) underwent variable clustering with the VARCLUS procedure using orthoblique rotation (JMP, version 14.0, SAS Institute, Cary, NC, USA), in which correlation among parameters was evaluated. In this method, a large set of variables can often be replaced by a set of cluster components automatically with little loss of information. The first principal component of each cluster was calculated, was the most representative variable, and was assigned a cluster score. The 12 acoustic parameters and the cluster cores were compared among the 4 groups using the non-parametric Kruskal-Wallis test, followed by the *post-hoc* Steel-Dwass test.

Mouse USVs have been classified by the shapes of the calls, by visualized in the spectrogram, and it was suggested that each call types carries some information (Neunuebel et al., 2015; Sangiamo et al., 2020). Call classification was conducted by k-means clustering using the abovementioned parameters, with the cluster number set as 9, based on previous studies (Kikusui et al., 2011; Sugimoto et al., 2011). The frequency of occurrence of these nine call types was compared among the four groups by MANOVA, followed by the Tukey-Kramer HSD *post-hoc* test.

Finally, the syllable transition was calculated for each mouse. We already reported that syllable transition is an important factor for the transfer of USV information from the sender to receiver (Takahashi et al., 2016). Inter-call intervals of more than 2 s were assigned as gaps due to inter-call intervals in call bouts being < 2 s (Takahashi et al., 2016). The frequency of the occurrences of the transition between the call was calculated. The average of the occurrences of the transition (more than 5%) in each experimental group was visualized in Figure 5.

## Results

### Acoustic Characteristics

Twelve acoustic parameters were compared among the 4 groups. Some group differences were found (Table 1, Supplementary Figure 1). Sex differences between control males and females were not observed. Similarly, there was no difference between TP-treated males and TP-treated females. TP-treated females showed higher frequency dynamics, delta frequency, mean frequency, and peak frequency as compared to control male and females (*p* < 0.05, Kruskal-Wallis test followed by Steel-Dwass test). TP-treated males showed higher mean frequency compared to control males and females (*p* < 0.05, Kruskal-Wallis test followed by Steel-Dwass test).

**Table 1:** The group comparisons of acoustic parameters.

	**Control**		**TP**	
	**Female**	**Male**	**Female**	**Male**
duration(ms):	44.49 ± 0.49	40.28 ± 0.32	47.76 ± 0.26	40.57 ± 0.26
freq dynamic(Hz)	8,680 ± 232^a^	9,492 ± 128^a^	14,386 ± 120^b^	9,774 ± 128
delta_Freq	457.7 ± 174.7^a^	435.2 ± 92.3^a^	5279.5 ± 89.4^b^	394.8 ± 126.4
meanFrequency(Hz)	54,941 ± 97^ab^	54,431 ± 59^a^	65,023 ± 61^c^	63,430 ± 72^cb^
frequencyTV(Hz)	27,861 ± 1,576^a^	26,817 ± 739	34,025 ± 691^b^	18,981 ± 450
meanFrequencyTV(Hz)	383.4 ± 17.0	414.6 ± 6.5	534.4 ± 6.6	354.1 ± 5.4
linearity index	4.76 ± 0.13	5.43 ± 0.08	7.74 ± 0.07	4.64 ± 0.07
nb modulation	1.15 ± 0.02	1.16 ± 0.02	1.42 ± 0.01	1.36 ± 0.01
nb jumps	0.12 ± 0.01	0.14 ± 0.01	0.21 ± 0.01	0.11 ± 0.01
minFrequency(Hz)	50,420 ± 133	49,854 ± 78^a^	58,001 ± 82^b^	58,552 ± 96^b^
maxFrequency(Hz)	59,100 ± 158	59,347 ± 100^a^	72,386 ± 92^b^	68,326 ± 106
peak Frequency(Hz)	55,563 ± 148^a^	54,977 ± 75^ab^	65,008 ± 73^c^	64,128 ± 85^ac^

*Values are Mean ± SEM*.

*Different alphabet letters (e.g., a and b) show statistical difference. Kruskal-Warris test, followed by Steel-Dwass test*.

### Clusters of Acoustic Characteristics

**Figure 1:**
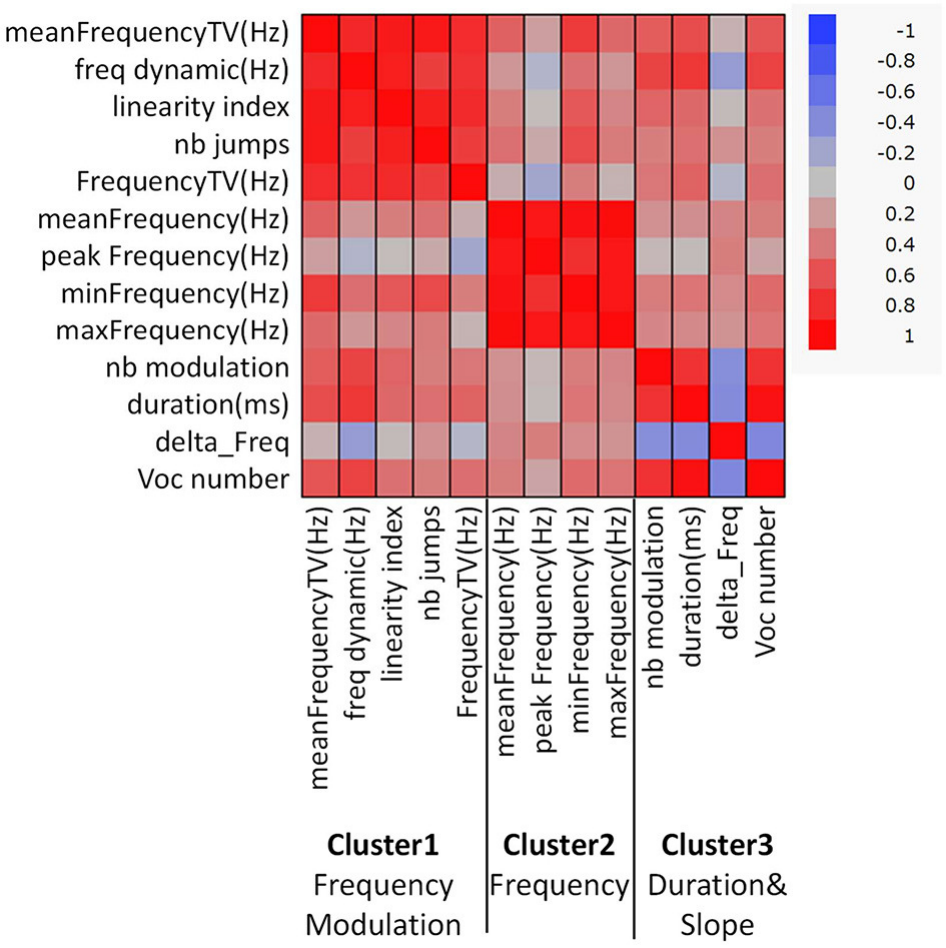
Correlation heatmap of 13 acoustic parameters and clustering with the VARCLUS procedure using orthoblique rotation (JMP, version 14.0, SAS Institute, Cary, NC, USA). Three representative clusters were obtained, as follows: Cluster 1, Frequency modulation; Cluster 2, Frequency; and Cluster 3, Duration and Slope.

**Figure 2:**
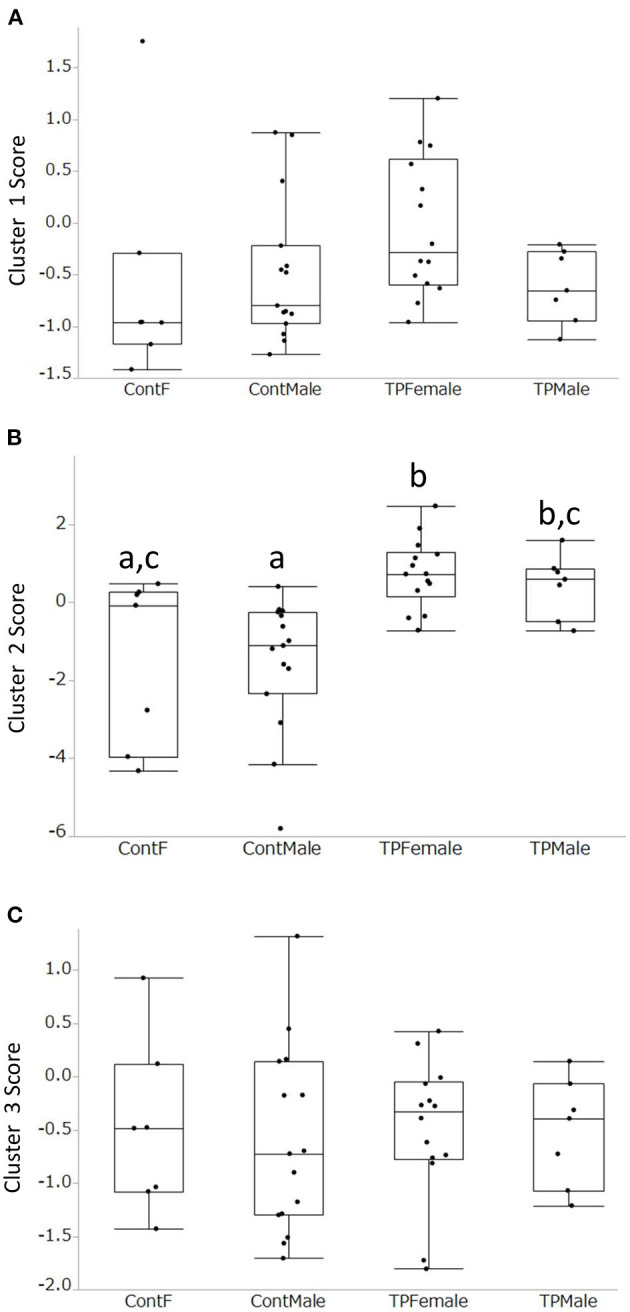
Comparisons of the 3 cluster scores among the 4 groups. There was no sex difference between control male and control female. **(A)**: Cluster 1, Frequency modulation; **(B)**: Cluster 2, Frequency; **(C)**: Cluster 3, Duration and Slope. In Cluster 2, TP-treated females had higher scores than control males and control females **(B)**. TP-treated males had higher scores than control males. Different alphabet letters (e.g., a and b) mean that there was a statistically significant difference between the two groups obtained using the Kruskal-Wallis test followed by the Steel-Dwass test.

Acoustic parameters were grouped into three clusters based on the correlations identified (Figure 1, Supplementary Table 2). Cluster 1 was composed of “mean frequency total variation (TV),” “frequency dynamic,” “linearity index,” “frequency TV,” and “number (nb) of jumps,” and these parameters were related to frequency modulation in calls. Cluster 2 was com-posed of “mean frequency,” “peak frequency,” “max frequency,” and “min frequency,” and these parameters were related to the frequency of the calls. Cluster 3 was composed of “nb modulation,” “duration,” “vocal (voc) number,” and “delta Frequency,” and these parameters were related to the duration and slope of the calls. The loading factors of each parameter are shown in Supplementary Table 2. There was a group difference in the Cluster 2 score (Figure 2, Supplementary Table 3, *p* < 0.05, *x*^2^ = 19.9, Kruskal-Wallis test), and scores of TP in Cluster 2 were higher than those of control male and control females (*z* = 3,81, *p* < 0.01; *z* = 2.64, *p* < 0.0.05, respectively, Steel-Dwass test). TP-treated males showed higher scores in Cluster 2 than the control males (*z* = 2.89, *p* < 0.05, Steel-Dwass test).

### Call Classification and Group Comparison

**Figure 3:**
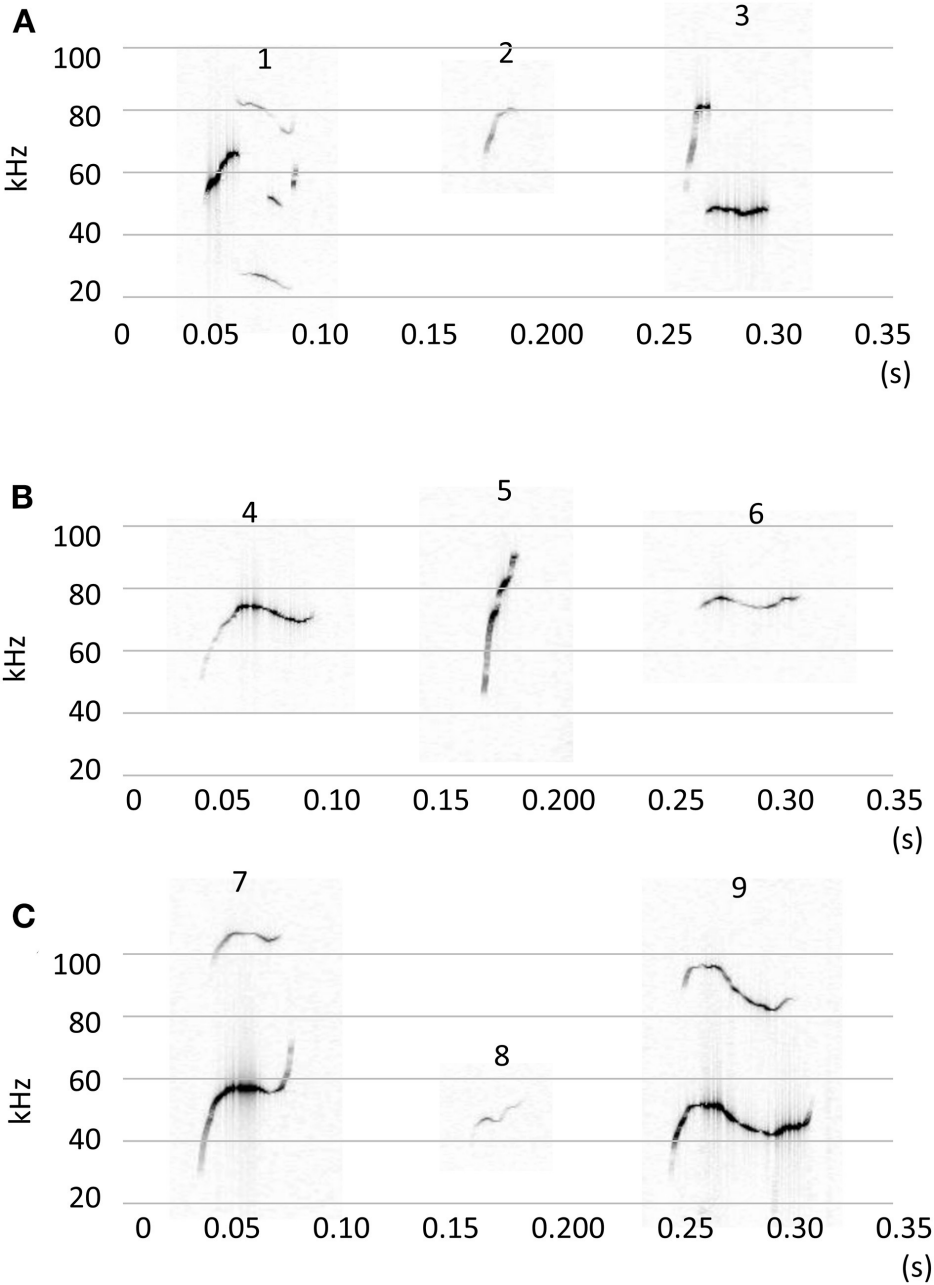
Representative figures of 9 call types **(A)** Call type 1–3. **(B)** Call type 4–6. **(C)** Call type 7–9. Calls were classified using 12 acoustic parameters by the k-mean clustering method.

**Figure 4:**
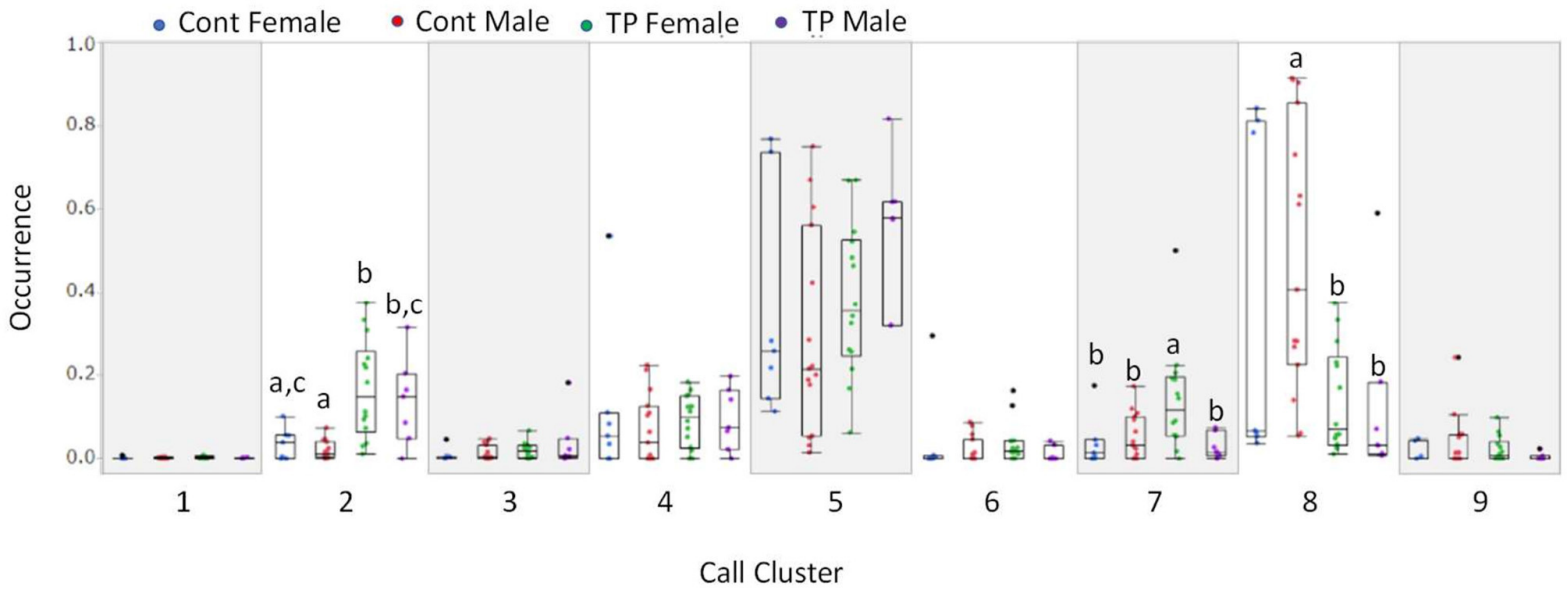
The probability of occurrence of 9 call types. The Y axis represents the probability of the occurrence of each call. Call types 2, 7, and 8 showed group differences.

Calls were classified into nine clusters according to 12 acoustic parameters (Figure 3). The acoustic parameters were averaged for each call type (Supplementary Table 4) and represented in standardization (z-score, Supplementary Figure 2). The following were the characteristics of the call types: Type 1, jump, high nb of jumps; Type 2, high frequency with short duration; Type 3, short segment with a flat segment; Type 4, long duration with a small slope; Type 5, slope with high frequency; Type 6, high linearly index and frequency of TV; Type 7, upward with frequency modulation; Type 8, high frequency with a short slope; and Type 9, long duration with modulations. The occurrences of each call type were calculated for each animal, and group comparisons were conducted using a multivariate analysis of variance (MANOVA). There were significant group differences [Figure 4; Roy's largest root =2.07, *F*_(10, 31)_ = 6.62, *p* < 0.00001]. In the *post-hoc* test, TP-treated females were associated with a higher occurrence of Type 2 calls than control females and control males (*p* < 0.05). TP-treated males were associated with a higher occurrence of Type 2 calls than control males (*p* < 0.05), and TP-treated females were associated with a higher occurrence of Type 7 calls than the other three groups (*p* < 0.05). Control males were associated with a higher occurrence of Type 8 calls compared to TP-treated females and TP-treated males (*p* < 0.05).

### Transition Probability

**Figure 5:**
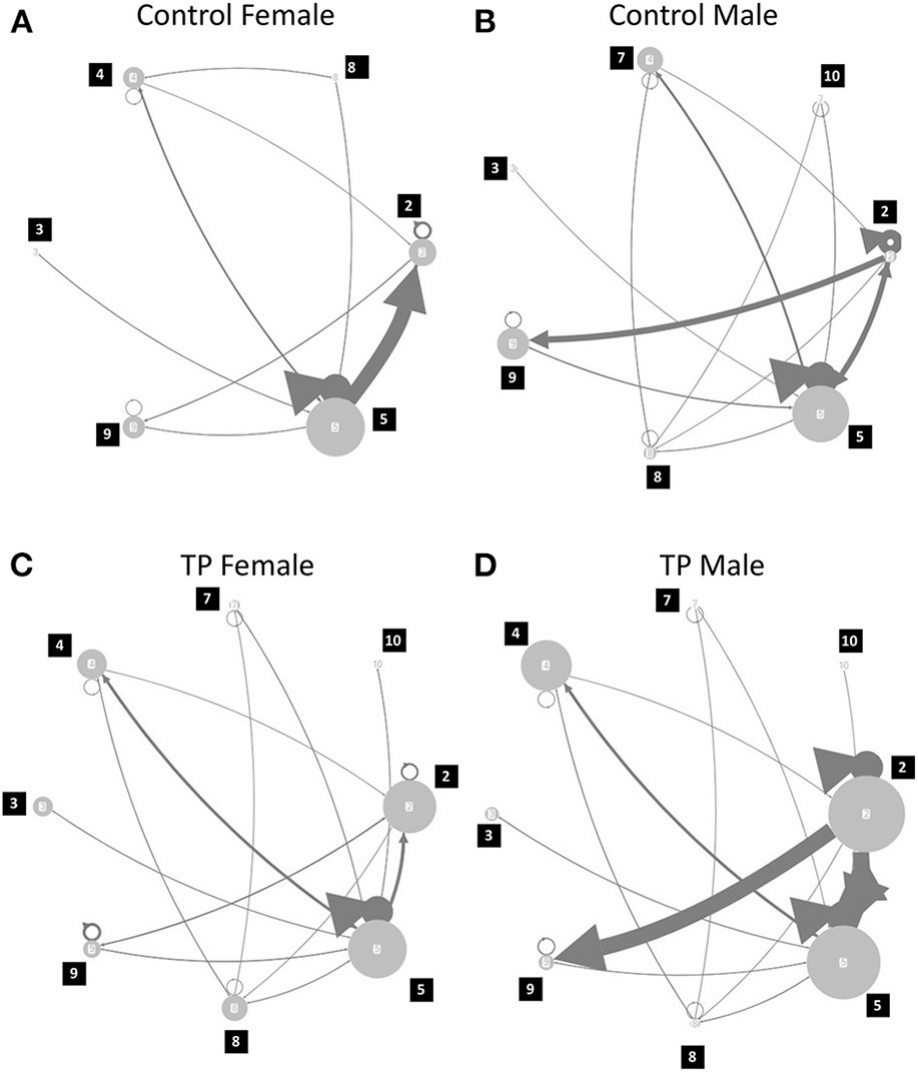
Call type transition probability. **(A)** Control Female; **(B)** Control Male; **(C)** TP female; **(D)** TP male. Numbers of nodes described the call types, and the width of the edge with arrows represented the probability of transition from one call type to another. For example, transition from type 2 calls to type 9 calls were obvious in control and TP-treated males, as compared to control and TP-treated females. There were self-transitions observed and it was represented with circles and triangles in each node (e.g., call type 2 and 5).

Finally, the call type transition was calculated for each mouse; the average transition probability is illustrated in Figure 5 (Supplementary Table 5). Although there were no statistical differences found, there was a tendency for sex differences; transition from Type 5 to 2 was highest in the control females, and the control males were associated with complex transitions and had a higher transition from Type 2 to 9. In TP-treated females, the transition pattern became complex, but a main transition from Type 5 to Type 2 was maintained in the control females. TP-treated males showed a similar pattern of transition to the control males; a high transition from Type 2 to 9.

## Discussion

In this study, we compared the acoustic characteristics of USVs between males and females, including TP-treated males and females. Perinatal and peripubertal testosterone treatment in females increased USV emissions compared to intact males, while the acoustic characteristics were not similar to those of intact males. Interestingly, there was no clear difference in acoustic characteristics between control males and females, suggesting that male and female emit USVs with similar acoustic characteristics, even though there was a difference in the number of calls. Previous reports described similar results, in which there were only minor differences in male and female USVs when encountering receptive females (Hammerschmidt et al., 2012; Neunuebel et al., 2015). When both male and female mice were treated with TP, they showed similar changes such as an increase in the frequency of calls. The complexity of the calls, such as the number of jumps, modulation, and duration of calls, were not different between males and females, including in the TP-treated males and females. TP can be aromatized into estradiol in the brain, suggesting that the frequency of calls was increased by exogenous androgenic and/or estrogenic stimulations in perinatal and peripubertal periods.

When the acoustic parameters were clustered, three clusters were obtained. The first cluster included frequency TV and frequency dynamics, indicating frequency modulation. The second cluster was composed of mean frequency and max frequency, and this was related to the call frequency. The third cluster comprised the duration of calls and the number of modulations, suggesting that this was related to call duration and slope. Interestingly, the number of calls in the test session was positively correlated with other parameters such as duration. This means that the more a mouse emitted USVs, the longer the duration of calls. The scores of the three clusters were compared and there was no obvious sex difference. Cluster 2 scores showed group differences; TP-treated females and males had higher scores, indicating that TP treatment in the perinatal and peripubertal periods increased the frequency of calls.

There are several ways to classify call types; we reported one in which the shapes, duration, and frequency of calls were visually determined and the calls were classified into 10 types (Kikusui et al., 2011). Using this method, we revealed strain differences in call types, which were mainly generated by the genetic background of mice (Kikusui et al., 2011). Several reports have presented the acoustic clustering of mouse USVs. For example, Hammerschmidt et al. demonstrated a two-step clustering method and identified three distinctive call types (Hammerschmidt et al., 2012). In this study, we used acoustic parameters to classify call types. We initially set the number of clusters to 10, as we have previously described (Kikusui et al., 2011). As a result, one cluster was identified as noise, and then nine call types were classified. The occurrence of type 2 calls with high frequency and short duration was higher in TP-treated females and male, contrary, type 8 calls were similar in shape as type 2 calls but with lower frequency and short duration, and the occurrence of type 8 calls was high in the control male and female. These were probably the reasons explain why TP-treated mice showed an acoustic characteristic of a higher frequency of calls. TP treatment did not modify the complex type of calls, such as types 1 and 9. These complex calls were frequently observed in mice during the approaching and moving away from counterparts in a large environment (50 × 50 cm or 76.2 × 76.2 cm) (Ey et al., 2020; Sangiamo et al., 2020), and these behaviors were difficult to observe in this experiment due to use of a standard cage. Therefore, if the testing area were larger, we could have observed the differences between males and female, and effect of TP treatment during perinatal and peripubertal periods in the occurrence of the call types. Recently, several statistical classifications were developed, revealing various call types (Ey et al., 2020; Sangiamo et al., 2020). An important point in these reports was that the occurrences of call types were social context dependent; a mouse, either male or female, can emit specific types of calls related to specific social behavior. For example, long and modulated calls were observed when the mouse showed close contact with another mouse (Ey et al., 2020). When a male mouse escapes from an aggressive male it emits calls with higher frequency, while males emit calls with lower frequency during aggressive episodes (Sangiamo et al., 2020). These results suggest that the occurrence of various call types reflects the emotional status of the subject mouse (Ey et al., 2020). We additionally analyzed anogenital sniffing behavior, which reflect sexual arousal, and found that the control female showed shorter duration of anogenital sniffing as compared to the other three groups (data not shown). The groups differences found in the acoustic characteristics were inconsistent with that found in anogenital sniffing. The testing area in this study comprised of a standard cage, and future studies are needed to clarify the effect of developmental TP treatment on the occurrence of the call types.

In this study, testosterone treatment increased the frequency of calls and modulated the occurrences of call types. However, the communicative functions of these changes were not yet revealed. Several studies revealed the correlation between occurrences of specific call types and recipient's behavior. In a male–female interaction in a large area, males and females synchronously emit USVs when males are chasing females, and the presence or absence of female calls could modulate the male-female interaction; female speed was significantly slower during chases with vocal interactions than without vocal interactions (Neunuebel et al., 2015). In four male mice groups, dominant calls from the chaser stimulated the speed of locomotion in the recipient males, indicating that the USV can convey emotional information from the emitter to modulate social behavior in the recipient (Sangiamo et al., 2020). We reported playback experiments in which male USVs were regenerated by the ultrasound emitter and observed the behavioral changes in females (Asaba et al., 2014; Nomoto et al., 2018, 2020). These reports revealed that females can recognize some specific characteristics of male USVs and show approaching behavior in response to the USVs. In the future, context-dependent and/or social behavior-dependent playback experiments are needed to clarify the social function of each call type, for example, changes of call types induced by testosterone treatment, in a complex social environment.

The pattern of transition of call types was not different between males and females. We reported that the transition patterns of male USVs were different among strains (Kikusui et al., 2011; Sugimoto et al., 2011). In addition, mouse pups' USVs also contained the specific call transition pattern, and the pattern was different between wildtype B6 and the genetic model of autism of TBX1 pups (Takahashi et al., 2016). If the call transition sequence was artificially randomized in B6 pup calls, the mother mouse did not show typical approach behavior to the randomized call sequences, even if the numbers of calls and types of calls were identical (Takahashi et al., 2016). These results suggest that the call type transition patterns are important for acoustic communication in mice. TP treatment in the perinatal and peripubertal periods was not effective in modulating the tendency of the difference between males and females, implying that the call type transition patterns are not modulated by hormones. Some reports showed that mouse *Sry* genes can be expressed in the central nervous system and modulate behavior in male mice (Dewing et al., 2006). Thus, even though there was no evidence of this in our study, it would be worth exploring this subject to reveal the function of sex-related genes in the central nervous system in relation to the pattern of transition of call types.

Sex differences in the acoustic characteristics of USVs are still controversial. In this study, we could not find any obvious sex differences in USV acoustic characteristics, which supports previous findings (Hammerschmidt et al., 2012; Neunuebel et al., 2015). However, Warren et al. revealed the following sex differences: the bandwidth and slope of vocal signals emitted by male and female mice were consistently different; female calls were narrower in bandwidth and females had more rapid changes in pitch than males (Warren et al., 2018). Differences in acoustic characteristics between male and female USVs may be due to the complexity of behaviors that the animals engage in as they vocalize, because the call characteristics were largely dependent on social contexts (Warren et al., 2018, 2020). Our study was conducted in a standard small cage and the behavioral variation was limited; therefore, it may have not been possible to detect sex differences in acoustic characteristics because of the behavioral testing context in the present study.

Several reports have demonstrated the emission of USVs by females in male-female interactions (Portfors, 2007; Neunuebel et al., 2015). In these reports, a pair of mice were introduced into a relatively large cage and they displayed a repertoire of complex behavior. To contrast, in the present study a pair of mice were in a small cage and USV emission by females might have been limited, as previously reported (Whitney et al., 1973; Warburton et al., 1989). However, we could not eliminate the possibility that the recorded USVs contained USVs emitted by the female mice. Indeed, the control females were encountered with sexually primed female opponents; thus, half of the USVs recorded in the control females could be from the counterpart subjects. The acoustic features of control females were composed of subject females and counterpart females, indicating that these calls were from two intact females. In other groups, the subject mice (control males, TP-treated males, and TP-treated females) showed intensive sniffing and approached the counterpart females, suggesting that these calls were mainly made by the subject mice as previously reported (Whitney et al., 1973; Warburton et al., 1989). While some recent papers showed female calls during male-female interaction (Portfors, 2007; Neunuebel et al., 2015), detected differences in the production of certain call types in the TP-treated subjects compared to control female-female subjects indicated that at least some of the calls emitted by the TP-treated subjects.

Another concern was the dose of TP injections. The acoustic features of TP-treated females and males were different to those of control males and females, suggesting that TP treatment did not mimic the physiological level of intact male pups. Therefore, one possibility was that the TP dose in this study was excessive when compared to the physiological levels in intact male pups. We previously reported the detailed time-dependent secretion pattern of testosterone in intact male and female pup brains (Mogi et al., 2015), but we do not yet know whether TP treatment can replicate the differences or not. Further analysis of testosterone and estrogen in the mouse brain after TP injections is needed to clarify this issue. In contrast, adult testosterone treatment has been shown to mimic testosterone secretion in intact males (Aoki et al., 2010). These perinatal and peripubertal treatments induced USVs and mounting behavior in females at a similar level to that seen in intact males, indicating that testosterone was sufficient to induce male sexual behavior, as previously reported (Aoki et al., 2010; Kikusui et al., 2020).

The neural mechanisms underlying the emission of USVs with complex call types, different frequency calls, and the pattern of transition of call types are yet to be uncovered. We previously reported that the neural circuits underscoring USV emissions and mounting behavior differ; the emission of USVs depends on the main olfactory circuit (Matsuo et al., 2015). Mice lacking the dorsal region of the main olfactory bulb demonstrated a decrease in USV emissions. We also previously reported clear sex differences in olfactory information processing in the medial amygdala, especially in the posterior regions (Kikusui et al., 2018). Genetic deletion of the vomeronasal neurons in females resulted in mounting behavior (Kimchi et al., 2007). In the targeted brain regions in the hypothalamus, several studied uncover the responsible nucleus for the production of USVs (Gao et al., 2019; Michael et al., 2020; Karigo et al., 2021). These results suggest that USV emissions depend on the main olfactory bulb-anterior olfactory nucleus-medial amygdala-hypothalamus. Further studies are needed to elucidate the temporal and spatial details of the androgenic/estrogenic modification of acoustic characteristics of USVs in the control of neural circuits.

## Data Availability Statement

The raw data supporting the conclusions of this article will be made available by the authors, without undue reservation.

## Ethics Statement

The animal study was reviewed and approved by Ethics Committee of Azabu University.

## Author Contributions

TK and KM: conceptualization. TK and KN: methodology. EE, FC, and TB: software. MS, YY, and TK: data collection. TK and MN: formal analysis. TK: writing—original draft preparation and funding acquisition. KM, KN, EE, FC, and TB: writing-review and editing. All authors have read and agreed to the published version of the manuscript.

## Conflict of Interest

The authors declare that the research was conducted in the absence of any commercial or financial relationships that could be construed as a potential conflict of interest.
