# Supplementary material for: Testosterone Increases the Emission of Ultrasonic Vocalizations With Different Acoustic Characteristics in Mice
Source: Front Psychol. 2021 Jun 25;12:680176. doi: 10.3389/fpsyg.2021.680176 (PMC8267093; doi:10.3389/fpsyg.2021.680176)
Supplement: Supplementary file 1 [file Data_Sheet_1.pdf]

Supplementary Material

1 Supplementary Figures and Tables

Supplementary Table S1.

Table S1. Definition of acoustic characters of USVs

Parameters	Definition
duration(ms):	Duration of each call, in millisecond.
freq dynamic(Hz)	Difference between the maximum and the minimum peak frequency of call.
delta_Freq	Difference between end Frequency and start Frequency
meanFrequency(Hz)	Mean of the peak frequencies over each timepoint in each call
FrequencyTV(Hz)	Total variation of the peak frequency for each t (sum of the absolute values of the difference between consecutive peak frequencies).
meanFrequencyTV(Hz)	FrequencyTVHz divided by the number of points in the USV.
linearity index	Sum of the euclidean distance to the linear regression divided by the number of points in the USV.
nb modulation	Number of times where the signal gets over and below the linear regression of the signal. Note that the signal should overtake linear regression by 876Hz (which corresponds to 3 times the frequency accuracy of the 1024 FFT)
nb jumps	The number of jumps is computed if the frequency dynamic of the USV is over 10 kHz. Then if the frequency changes from one t to the next over than ⅓ of the frequency dynamic, we set one jump.
minFrequency(Hz)	Minimum peak frequency
maxFrequency(Hz)	Maximum peak frequency
peak Frequency(Hz)	Peak frequency (highest) in each call

(cited from Ey. et al. 2021)

Supplementary Table S2

Table S2. Factor Loardings of 13 acoustic parameters on 3 Clusters

Parameters	Cluster1	Cluster2	Cluster3
meanFrequencyTV(Hz)	0.467		
freq dynamic(Hz)	0.465		
linearity index	0.438		
frequencyTV(Hz)	0.437		
nb jumps	0.427		
meanFrequency(Hz)		0.514	
peak Frequency(Hz)		0.511	
maxFrequency(Hz)		0.489	
minFrequency(Hz)		0.486	
nb modulation			0.559
duration(ms):			0.554
voc number			0.514
delta_Freq			-0.342

Supplementary Table S3

Table S3. Cluster scores of each group

	Control		TP	
	Female	Male	Female	Male
Cluster1	0.646 ± 0.035	-0.037 ± 0.017	-0.005 ± 0.016	-0.370 ± 0.014
Cluster2	0.053 ± 0.021 [ac]	-0.906 ± 0.013 [a]	0.375 ± 0.013 [b]	0.826 ± 0.016 [bc]
Cluster3	0.258 ± 0.019	0.101 ± 0.013	-0.081 ± 0.011	-0.202 ± 0.011

values are mean ± sem

Different alphabet letters (ex. a and b) show statistical difference. Kruskal-Warris test, followed by Steel-Dwass test.

Supplementary Table S4

Table S4-1. Acoustic characters of 9 clusters of call types: MeanValues

	1	2	3	4	5	6	7	8	9
duration(ms):	79.32	46.43	67.33	89.76	38.37	78.71	73.16	36.15	139.94
freq dynamic(Hz)	33654	11314	37535	11624	5921	47080	39210	5443	45527
delta_Freq	-1537	2950	-28728	-3048	3288	-6625	7067	2159	-7580
meanFrequency(Hz)	60531	70407	62009	60185	61143	63856	61161	48729	58355
FrequencyTV(Hz)	142721	19939	77051	27651	10264	298604	120967	9099	319812
meanFrequencyTV(Hz)	1667.3	370.1	1062.3	275.0	225.2	3302.5	1518.8	208.7	2099.9
linearity index	17.411	5.587	17.739	6.236	2.532	25.094	21.179	2.611	26.217
nb modulation	3.071	1.743	1.664	2.551	1.234	2.120	1.661	0.979	8.123
nb jumps	5.176	0.115	0.357	0.115	0.017	0.231	0.584	0.036	0.311
minFrequency(Hz)	45080	64716	44487	54588	57903	39676	41310	45867	36923
maxFrequency(Hz)	78735	76030	82022	66212	63824	86755	80520	51311	82450
peak Frequency(Hz)	60107	71185	61382	61773	61986	64275	62058	49077	57417

Table S4-2. Acoustic characters of 9 clusters of call types: Standard deviation

	1	2	3	4	5	6	7	8	9
duration(ms):	34.66679	16.58666	25.70891	29.83539	13.777	18.80476	27.22923	19.01855	45.70292
freq dynamic(Hz)	8452.288	6719.612	9889.423	5145.226	3404.143	5893.949	6661.878	4170.336	8306.492
delta_Freq	12661.09	9318.883	9766.336	6864.363	4607.201	17715.22	13560.33	4885.214	12795.09
meanFrequency(Hz)	7031.541	5761.734	7113.996	3889.582	2916.086	6119.934	7130.845	7234.677	5644.81
FrequencyTV(Hz)	69603.59	12332.31	49382.91	12238.9	5148.771	85729.58	52059.53	7433.232	126314.9
meanFrequencyTV(Hz)	834.798	220.19	614.8692	114.8626	92.72811	842.8254	608.711	130.6515	842.4457
linearity index	6.567797	4.126065	8.30385	2.976599	1.729604	4.593016	5.653118	2.637074	7.486638
nb modulation	2.127561	0.910041	1.221077	0.795689	0.955144	1.63843	1.473947	1.013357	2.862466
nb jumps	2.141024	0.383604	0.610441	0.391074	0.140692	0.570403	0.818614	0.213116	0.691994
minFrequency(Hz)	6856.405	6387.282	11165.91	4624.67	3533.707	4046.008	7040.535	6642.226	4449.251
maxFrequency(Hz)	7690.025	7167.466	9890.56	5416.018	3408.948	6567.513	9005.355	8125.069	9035.545
peak Frequency(Hz)	12125.19	6460.679	11554.74	4407.235	3060.018	16818.18	12072.44	7635.688	12479.46

Supplementary Table S5

Table S5. Probability (%) of transition of call types in each group

		Control		TP	
Vertex 1	Vertex 2	Female	Male	Female	Male
1	1	0.00	0.00	0.00	0.00
1	2	0.00	0.00	0.00	0.00
1	3	0.00	0.00	0.00	0.00
1	4	0.00	0.00	0.00	0.00
1	5	0.00	0.00	0.00	0.00
1	6	0.00	0.00	0.00	0.00
1	7	0.00	0.00	0.00	0.00
1	8	0.00	0.00	0.00	0.00
1	9	0.00	0.00	0.00	0.00
2	1	0.00	0.00	0.00	0.00
2	2	4.57	12.89	3.54	10.69
2	3	0.00	0.00	0.00	0.00
2	4	0.00	0.00	0.00	0.00
2	5	5.27	7.93	4.16	12.40
2	6	0.00	0.00	0.00	0.00
2	7	0.00	0.29	0.40	0.17
2	8	2.62	9.20	2.74	12.15
2	9	0.00	0.00	0.00	0.00
3	1	0.00	0.00	0.00	0.00
3	2	0.00	0.00	0.00	0.00
3	3	0.00	0.00	0.00	0.00
3	4	0.00	0.00	0.00	0.00
3	5	0.49	0.31	0.59	0.27
3	6	0.00	0.00	0.00	0.00
3	7	0.00	0.00	0.00	0.00
3	8	0.00	0.00	0.00	0.00
3	9	0.00	0.00	0.00	0.00
4	1	0.00	0.00	0.00	0.00
4	2	0.38	0.28	0.42	0.18
4	3	0.00	0.00	0.00	0.00
4	4	1.15	1.41	1.55	0.76
4	5	2.88	3.45	4.28	1.88
4	6	0.00	0.00	0.00	0.00
4	7	0.36	0.51	0.45	0.26
4	8	0.00	0.00	0.00	0.00
4	9	0.00	0.00	0.00	0.00
5	1	0.00	0.00	0.00	0.00
5	2	19.97	7.28	5.33	12.36
5	3	0.51	0.00	0.35	0.22
5	4	3.19	3.85	4.57	2.10
5	5	52.80	40.66	54.55	40.74
5	6	0.00	0.27	0.28	0.00
5	7	0.68	1.00	1.21	0.48
5	8	1.65	1.29	1.77	0.68
5	9	0.00	0.00	0.26	0.00
6	1	0.00	0.00	0.00	0.00
6	2	0.00	0.00	0.00	0.00
6	3	0.00	0.00	0.00	0.00
6	4	0.00	0.00	0.00	0.00
6	5	0.00	0.31	0.30	0.14
6	6	0.00	0.43	0.41	0.19
6	7	0.00	0.00	0.00	0.00
6	8	0.00	0.00	0.00	0.00
6	9	0.00	0.00	0.00	0.00
7	1	0.00	0.00	0.00	0.00
7	2	0.00	0.00	0.00	0.00
7	3	0.00	0.00	0.00	0.00
7	4	0.40	0.51	0.69	0.25
7	5	0.75	1.08	1.27	0.55
7	6	0.00	0.27	0.28	0.14
7	7	0.00	0.62	0.77	0.30
7	8	0.00	0.00	0.00	0.00
7	9	0.00	0.00	0.00	0.00
8	1	0.00	0.00	0.00	0.00
8	2	0.00	0.81	2.42	1.03
8	3	0.00	0.00	0.00	0.00
8	4	0.00	0.00	0.00	0.00
8	5	1.76	2.79	2.37	0.69
8	6	0.00	0.00	0.00	0.00
8	7	0.00	0.00	0.00	0.00
8	8	0.58	2.55	4.79	1.26
8	9	0.00	0.00	0.00	0.00
9	1	0.00	0.00	0.00	0.00
9	2	0.00	0.00	0.00	0.00
9	3	0.00	0.00	0.00	0.00
9	4	0.00	0.00	0.00	0.00
9	5	0.00	0.00	0.27	0.13
9	6	0.00	0.00	0.00	0.00
9	7	0.00	0.00	0.00	0.00
9	8	0.00	0.00	0.00	0.00
9	9	0.00	0.00	0.00	0.00

Values are mean of % of occurrence of transition

Supplementary Figure S1

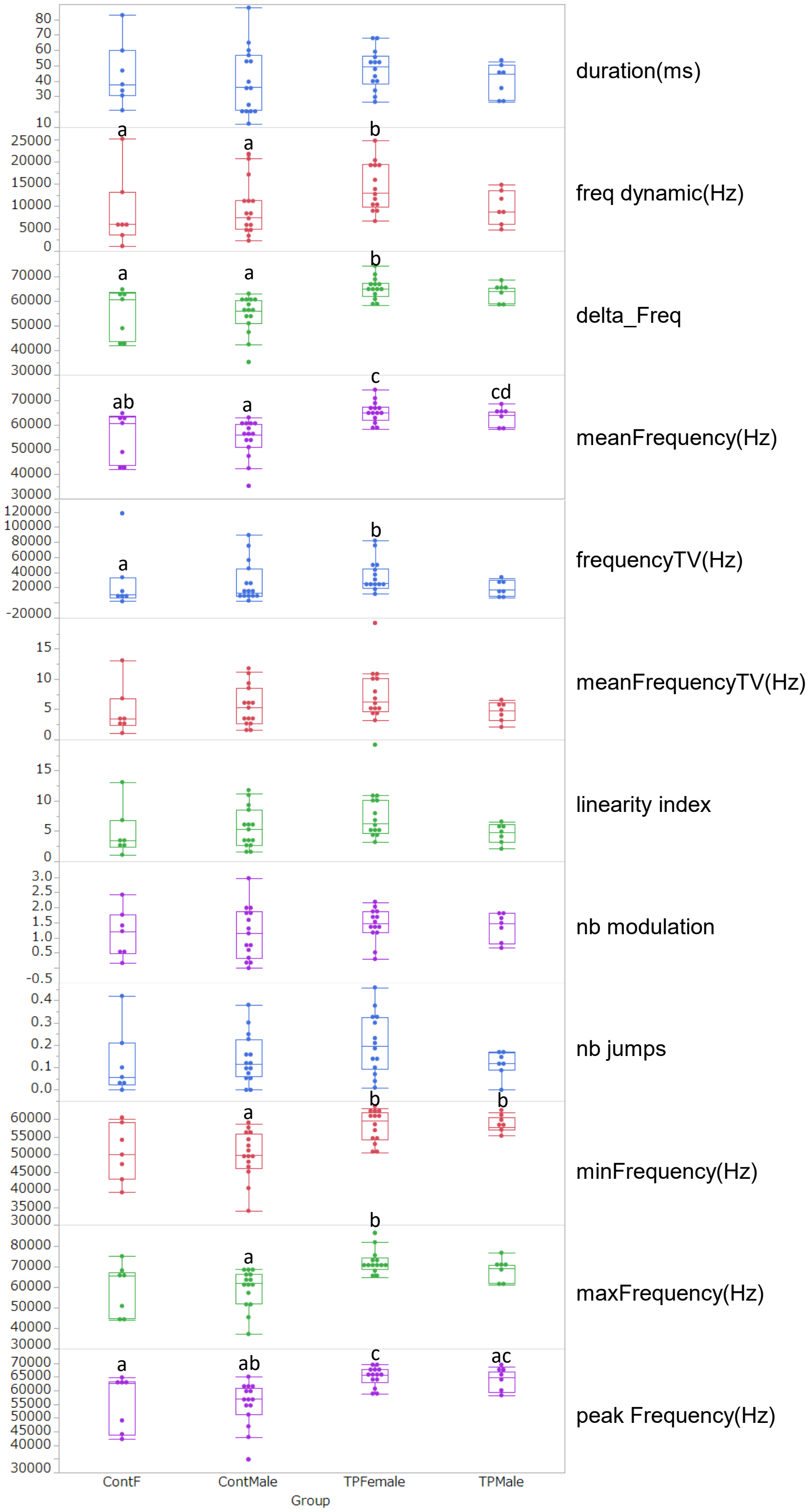

Supplementary Figure 1. Twelve acoustic parameters were compared among the 4 groups. Different letter alphabet letters (e.g., a and b) mean that there was a statistically significant difference between the two groups obtained using the Kruskal-Wallis test followed by the Steel-Dwass test.

Supplementary Figure S2

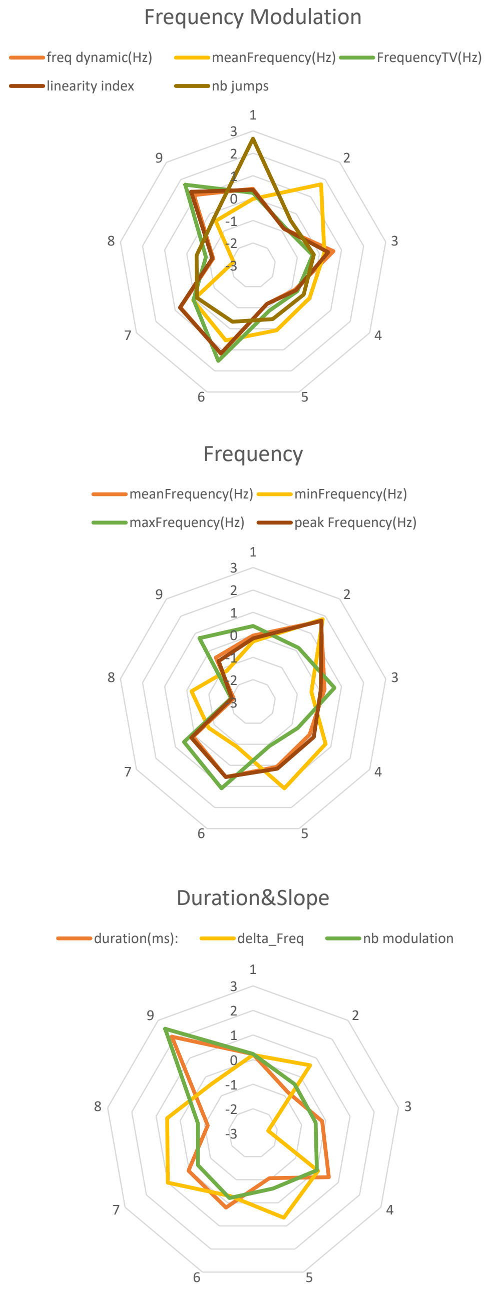

Supplementary Figure 2. Acoustic characteristics of the 9 call types. The acoustic parameters were grouped in 3, according to the clustering result (Figure 1), and each call types (1 to 9) were plotted in the radar charts (z scores).
